# Supplementary material for: Mirusviruses link herpesviruses to giant viruses
Source: Nature. 2023 Apr 19;616(7958):783–9. doi: 10.1038/s41586-023-05962-4 (PMC10132985; doi:10.1038/s41586-023-05962-4)
Supplement: Supplementary file 1 — Reporting Summary [file 41586_2023_5962_MOESM1_ESM.pdf]

nature portfolio

Corresponding author(s): Tom Delmont

Last updated by author(s): Mar 5, 2023

Reporting Summary

Nature Portfolio wishes to improve the reproducibility of the work that we publish. This form provides structure for consistency and transparency in reporting. For further information on Nature Portfolio policies, see our Editorial Policies and the Editorial Policy Checklist.

Statistics

For all statistical analyses, confirm that the following items are present in the figure legend, table legend, main text, or Methods section.

n/a	Confirmed	
☐	☒	The exact sample size (*n*) for each experimental group/condition, given as a discrete number and unit of measurement
☒	☐	A statement on whether measurements were taken from distinct samples or whether the same sample was measured repeatedly
☐	☒	The statistical test(s) used AND whether they are one- or two-sided<br>*Only common tests should be described solely by name; describe more complex techniques in the Methods section.*
☒	☐	A description of all covariates tested
☒	☐	A description of any assumptions or corrections, such as tests of normality and adjustment for multiple comparisons
☒	☐	A full description of the statistical parameters including central tendency (e.g. means) or other basic estimates (e.g. regression coefficient) AND variation (e.g. standard deviation) or associated estimates of uncertainty (e.g. confidence intervals)
☐	☒	For null hypothesis testing, the test statistic (e.g. *F*, *t*, *r*) with confidence intervals, effect sizes, degrees of freedom and *P* value noted<br>*Give P values as exact values whenever suitable.*
☒	☐	For Bayesian analysis, information on the choice of priors and Markov chain Monte Carlo settings
☒	☐	For hierarchical and complex designs, identification of the appropriate level for tests and full reporting of outcomes
☒	☐	Estimates of effect sizes (e.g. Cohen's *d*, Pearson's *r*), indicating how they were calculated

Our web collection on statistics for biologists contains articles on many of the points above.

Software and code

Policy information about availability of computer code

Data collection	no software was used to collect data
Data analysis	Genome-resolved metagenomics and visualizations were done using the platform anvi'o (v.7). HMMER (v3.1b2) was used to run Hidden Markov models. CD-HIT (v4.8.1) was used to remove protein redundancies. In order to perform phylogenetic analyses, we used MAFFT v7.464, Goalign v0.3.5, and IQ-TREE v1.6.2. Hallmark gene curations were performed using BLAST (v2.10.1). Metagenomic and metatranscriptomic read recruitments (mappping) were done using BWA v0.7.15. Both Orthofinder (v2.5.2), AGNOSTOS (v.1), and Linclust using MMseqs (v13-45111) were used to generate gene and protein clusters. Functional annotations were done using Pfam v35, PDB70, and UniProt/Swiss-Prot viral protein databases. They were also done using Virus-Host DB, RefSeq, UniRef90, NCVOGs (updated to the November 2021 version), and the NCBI nr database (August 2020), with using Diamond (v2.0.6). In addition, KEGG Orthology and functional categories were assigned with the Eggnog-Mapper (v2.1.5), and tRNAscan-SE75 (v2.0.7) was used to predict tRNAs. 3D structures were modeled using AlphaFold2 (v2.3.0) and RoseTTAFold v1.4. Foldseek (v4.64577) was used to compare protein 3D structures.

For manuscripts utilizing custom algorithms or software that are central to the research but not yet described in published literature, software must be made available to editors and reviewers. We strongly encourage code deposition in a community repository (e.g. GitHub). See the Nature Portfolio guidelines for submitting code & software for further information.

Data

Policy information about availability of data

All manuscripts must include a data availability statement. This statement should provide the following information, where applicable:

- Accession codes, unique identifiers, or web links for publicly available datasets
- A description of any restrictions on data availability
- For clinical datasets or third party data, please ensure that the statement adheres to our policy

Databases our study used include (1) the TARA Oceans metagenomes and metatranscriptomes (https://www.ebi.ac.uk/ena/browser/view/PRJEB402), (2) publicly available Nucleocytoviricota MAGs (https://www.nature.com/articles/s41586-020-1957-x and https://www.nature.com/articles/s41467-020-15507-2), (3) and Virus-Host DB (https://www.genome.jp/virushostdb/), (4) RefSeq (https://ftp.ncbi.nlm.nih.gov/refseq/), (5) UniRef90 (https://ftp.ebi.ac.uk/pub/databases/uniprot/uniref/uniref90/), (6) NCVOG (https://ftp.ncbi.nih.gov/pub/wolf/COGs/NCVOG/) and (7) NCBI nr database (https://ftp.ncbi.nih.gov/blast/db/). Data our study generated has been made publicly available at https://doi.org/10.6084/m9.figshare.20284713. This link provides access to (1) the RNApolB genes reconstructed from the Tara Oceans assemblies (along with references), (2) individual FASTA files for the 1,593 non-redundant marine Nucleocytoviricota and mirusvirus MAGs (including the 697 manually curated MAGs from our survey) and 224 reference Nucleocytoviricota genomes contained in the GOEV database, (3) the GOEV anvi'o CONTIGS database, (4) genes and proteins found in the GOEV database, (5) manually curated hallmark genes, (6) predicted 3D structures of the Duplodnaviria virion module (includes proteins and their alignments), (7) phylogenies and associated anvi'o PROFILE databases with metadata, (8) HMMs for hallmark genes, (9) a FASTA file for the near-complete contiguous genome (SAMEA2619782_METAG_scaffold_2), (10) and all the supplemental tables.

Human research participants

Policy information about studies involving human research participants and Sex and Gender in Research.

Reporting on sex and gender	Not applicable
Population characteristics	Not applicable
Recruitment	Not applicable
Ethics oversight	Not applicable

Note that full information on the approval of the study protocol must also be provided in the manuscript.

Field-specific reporting

Please select the one below that is the best fit for your research. If you are not sure, read the appropriate sections before making your selection.

☐ Life sciences ☐ Behavioural & social sciences ☒ Ecological, evolutionary & environmental sciences

For a reference copy of the document with all sections, see nature.com/documents/nr-reporting-summary-flat.pdf

Ecological, evolutionary & environmental sciences study design

All studies must disclose on these points even when the disclosure is negative.

Study description	The study is based on metagenomic data generated by the Tara Oceans consortium over the years. We characterized and manually curated environmental genomes for giant viruses as well as a previously unknown clade dubbed "Mirusviricota".
Research sample	Sunlit oceans (plankton)
Sampling strategy	The study did not involve any sampling, and we used data generated by the Tara Oceans consortium
Data collection	We used all Tara Oceans metagenomes. Those were generated in our institute s part of previous publications, so we had direct access to the data. The data is also publicly available to others.
Timing and spatial scale	The study did not involve any sampling or other data collection.
Data exclusions	No data was excluded.
Reproducibility	All data is available, and the tool anv'io is available for all to reproduce our findings.
Randomization	We worked on all the metagenomic legacy of Tara Oceans, so their is no randomization.
Blinding	We worked on all the metagenomic legacy of Tara Oceans, so their is no blinding.

Did the study involve field work? ☐ Yes ☒ No

Reporting for specific materials, systems and methods

We require information from authors about some types of materials, experimental systems and methods used in many studies. Here, indicate whether each material, system or method listed is relevant to your study. If you are not sure if a list item applies to your research, read the appropriate section before selecting a response.

Materials & experimental systems

n/a	Involved in the study
☒	☐ Antibodies
☒	☐ Eukaryotic cell lines
☒	☐ Palaeontology and archaeology
☒	☐ Animals and other organisms
☒	☐ Clinical data
☒	☐ Dual use research of concern

Methods

n/a	Involved in the study
☒	☐ ChIP-seq
☒	☐ Flow cytometry
☒	☐ MRI-based neuroimaging
